# A novel press-fit minimally-invasive symphysiodesis technique

**DOI:** 10.1186/s40634-020-00284-0

**Published:** 2020-09-17

**Authors:** Sascha J. Hopp, Antonius Pizanis, Jeremy Briem, Jill Hahner, Laura Mettelsiefen, Steven C. Herath, Tina Histing, Tim Pohlemann, Tobias Fritz

**Affiliations:** 1 Department of Trauma, Hand and Reconstructive Surgery, University Hospital of Saarland, Kirrbergerstr 1, 66421 Homburg/Saar, Germany; 2 Groin Pain and Core Injury Center, Lutrina Clinic, Karl-Marx-Straße 33, 67655 Kaiserslautern, Germany

**Keywords:** Pelvis, Pubic Symphysis, Symphysiodesis, Arthrodesis Symphysis, Non-union Symphysis

## Abstract

**Objective:**

Instability of the pubic symphysis often results in a poor outcome and reduced mobility of the patient. In some cases, an arthrodesis of the pubic symphysis is required. Until today, there is no data published how many of these procedures are performed annually and there is also no data about the outcome after this extensive surgery.

**Methods:**

We developed a novel surgical technique to address the arthrodesis of the pubic symphysis in a minimally invasive approach. Therefore, we used for this purpose modified instruments and performed the transplantation of a cylindrical bone substitute into the pubic symphysis, without an extensive approach or dissecting the anterior or posterior symphyseal ligaments.

**Results:**

Using this novel technique, a minimally invasive symphysiodesis was achieved in radiological findings, after the procedure.

**Conclusion:**

Thus, this actually minimally invasive surgical technique seems to be a promising advancement for the arthrodesis of the pubic symphysis.

## Introduction

Chronic anterior pelvic ring instability has been associated with chronic pain and often results in poor patient outcome [1].

The instability of the pubic symphysis often occurs posttraumatically, for example after a symphyseal rupture, related to pelvic ring injuries [1–3]. Chronic instability can also result from non-traumatic conditions like osteitis pubis [1] or chronic sportive overuse with repeated adductor tendon injuries [4]. Furthermore, osteitis pubis resulting from rheumatologic disorders and puerperal symphyseal rupture after delivery have been reported within 1 in 300 to 1:30000 cases [1, 5–7]. The treatment of these patients often is challenging and implant failure after plate stabilization is well described in the literature. However, these complications are usually tolerated by most authors [1]. For the treatment of non-traumatic symphyseal ruptures, there are only few reported cases [1], but there are still some complex cases, in which even plate stabilization doesn’t result in pain free walking. For these specific cases, an arthrodesis of the pubic symphysis (symphysiodesis) has been advocated, with promising data of the published cases [1, 8–10]. For this technique, usually an open modified Pfannenstiel approach is used. The anterior, posterior and cranial ligaments of the symphysis are dissected and the bony parts of the pubic symphysis are removed on both sides. Subsequently, a tricortical autologous bone graft from the iliac crest is harvested and transplanted into the prepared area. Usually the symphysiodesis is stabilized using one cranial plate or a cranial plate with an additional “bumper” plate [8, 11]. Because this surgical procedure can result in approach related complications such as wound infection or prolonged healing, we established a novel surgical technique, which can be performed minimally-invasive. Therefore, existing instruments were modified in order to be in a position to perform a minimally-invasive symphysiodesis using a cylindrical bone substitute on a human specimen without further dissecting the stabilizing anterior or posterior symphyseal ligaments. To our knowledge, this is the first attempt to address symphyseal instability using a press-fit cylindrical bone substitute for arthrodesis. In the future, we presume that chronic instability of the pubic symphysis can be reliably treated using this minimally invasive technique in addition to our published minimally invasive stabilization technique of the pubic symphysis using an internal fixator [2] or even without osteosynthesis for stabilization.

## Material & Methods

### Instruments

**Fig. 1:**
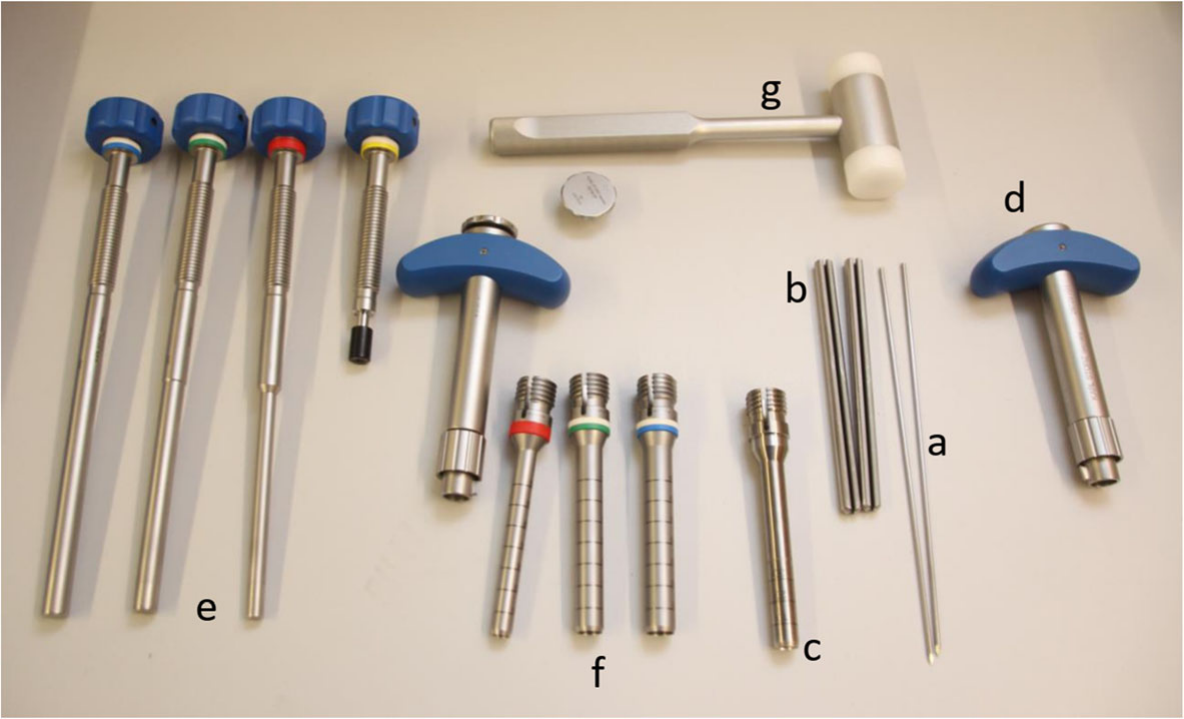
The complete selection of instruments for the autologous transplantation of a bone cylinder. **a** Guidewire, **b** novel centering device for the bone harvester, **c** novel, modified bone harvester with centering fins with an inner diameter of 10 mm, **d** handle, **e** harvested bone remover, **f** bone harvester with an inner diameter of 10, 12, 14 mm, **g** hammer

For this technical protocol, a novel additional guide sleeve, for the already existing Bone Block Harvesting system, was created in collaboration with KARL STORZ (Tuttlingen, Germany) (Fig. 1). To fit the inner diameter of 8 mm and to allow for a sufficient guidance, two fins at the inferior end were created. The guide sleeve itself also was cannulated to allow the placement of a guidewire prior to the placement of the guidewire sleeve.

## Specimens

We investigated on one fixated complete human female cadaver. The cadaver had no history of pelvic ring fractures or pelvic ring instability.

## Surgical procedure

The human specimen was positioned in supine position on a radiolucent table. Before the skin incision, fluoroscopy was used in anterior-posterior, inlet and outlet position to gain adequate orientation during procedure.

Following these preparations, a scalpel blade was placed at the top of the pubic symphysis in both inlet and outlet view. After that, a transverse skin incision of 2 cm was performed. The subcutaneous tissue was dissected by palpating the bony pubic symphysis superior and then spreading the tissue using scissors.

**Fig. 2:**
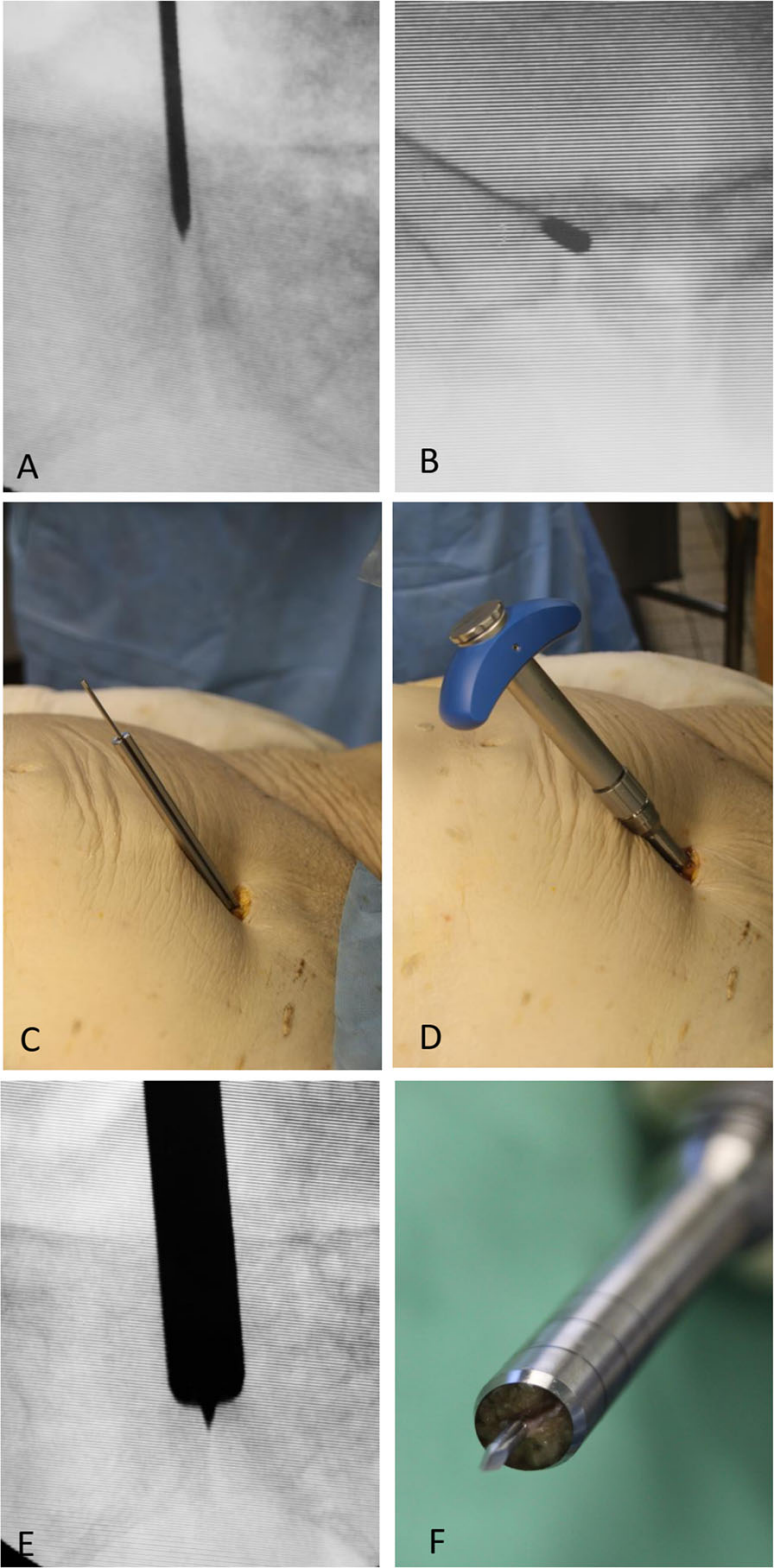
**a** and **b**: Placement of the guidewire inside the pubic symphysis using inlet & outlet radiographs **c** Using the centering device across the guidewire and pushing it down onto the bony part of the symphysis **d** Using the novel modified bone harvester with fins, that is centered and driving it into the bone using a hammer **e** Inlet-radiograph, showing the correct positioning of the bone harvester, also leaving the caudal part and the caudal ligaments of the pubic symphysis intact **f** The harvested pubic symphysis inside the bone harvester with the guidewire inside the pubic symphysis

**Fig. 3:**
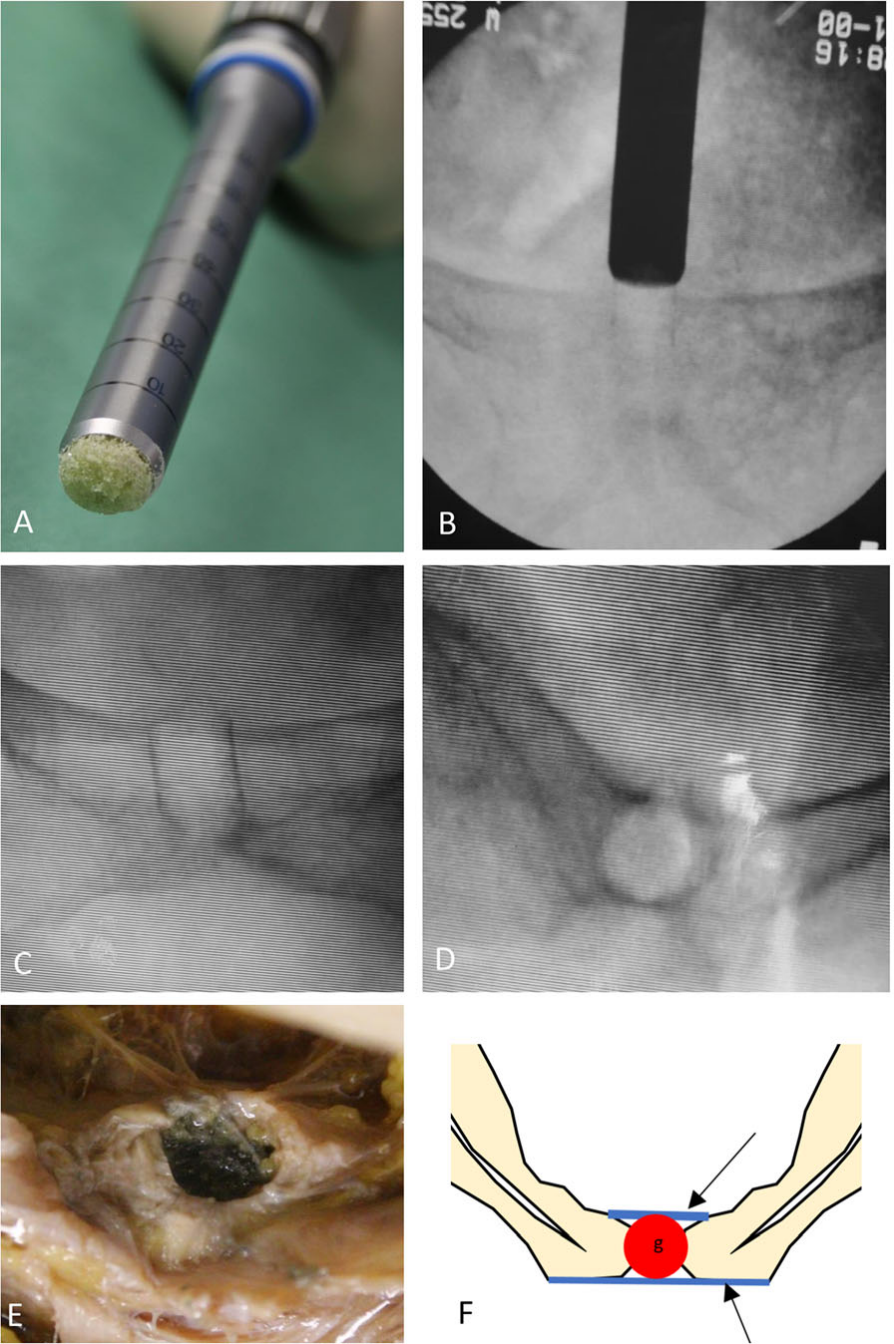
**a** The Synbone cylinder for transplantation inside the harvesting device. **b** Radiograph showing the Synbone cylinder being pushed press-fit into the pubic symphysis **c** & **d**: Radiograph showing the transplanted cylinder inside the pubic symphysis **e** After dissection of the cranial part of the soft tissue shows remaining anterior and posterior ligaments of the pubic symphysis. **f** Schematic drawing of the pubic symphysis with the ligaments anterior and posterior still intact with the bone cylinder press-fit inside the pubic symphysis

Next, a 2.0 mm guide wire was positioned inside the pubic symphysis, using a motor drill and fluoroscopy in inlet and outlet position verified its central position (Fig. 2a, b). After correct placement of the guide wire, the guide probe was placed over it to achieve a central positioning (Fig. 2c). Then the bone harvesting instrument was driven 3 cm deep into the pubic symphysis (Fig. 2d, e) in order to remove a 10 mm bone-symphysis-bone cylinder (Fig. 2f). After that, the cylinder created (diameter 10 mm x length 30 mm) and the guide wire were removed. A cylinder (diameter 13 mm x length 30 mm) made out of bone substitute (Synbone, Foam Block, 5 PCF, Zizers, Switzerland) was prepared, using the novel bone harvesting instrument (KARL STORZ, Tuttlingen, Germany) (Fig. 3a). The bone substitute was impregnated using Zinc-paint to increase visibility while using fluoroscopy and for later anatomical dissection for study purposes. Subsequent to this, the cylinder was placed at the artificial opening superior at pubic symphysis and then successively introduced with light hammer blows into the created cavity using fluoroscopic guidance (Fig. 3b). The final fluoroscopic imaging showed no signs of displacement of the pelvic ring and a proper press-fit position of the bone substitute within the bony confines of the pubic symphysis (Fig. 3c, d).

### Anatomical dissection after the procedure

After completing the surgical procedure, we dissected the pubic symphysis to analyze the correct position and the perisymphyseal ligaments (Fig. 3e). There were no signs of ligament insufficiency in the specimen. The implanted bone substitute showed a central positioning in both anterior-posterior and inferior-superior axis as shown in the drawing (Fig. 3f).

## Discussion

Chronic instability of the pubic symphysis may result from inadequate posttraumatic healing of the symphyseal ligaments, degenerative changes due to chronic sportive overuse and adductor tendon injuries [4], osteitis pubis [10] or post-partum because of hormonal misbalance [8, 9]. These changes often result in persisting pain and may cause severe immobility. In an early stage, a revised stabilization of the pubic symphysis using plate fixation or novel minimally invasive methods may provide enough stability for ligamentous healing [10]. However, there are still patients with persisting chronic instability, which results in a high demand of pain medication and often poor life-quality. In such cases, arthrodesis of the pubic symphysis (symphysiodesis) often is the last option to achieve adequate stability [1, 8]. Pohlemann & Tscherne described the implantation of a tricortical autologous bone block from the iliac crest, which was modified by Giannoudis et al. using a T-shaped bone graft [8, 11]. To stabilize the bone block and the symphysis pubis, usually a cranial 4.5 dynamic compression plate (DCP) or 3.5 symphyseal locking plate (SLDCP) often in combination with a second anterior “bumper” plate are used [8, 11, 12]. Although the surgical technique has been described over 20 years ago, only few specialists treat a little number **of** cases per year and no data about complication rates or the outcome after that procedure have been published [13]. An extensive surgical approach, the modified Pfannenstiel incision, usually is used for this technique. Considering this, local regional pain syndromes (Pfannenstiel syndrome) [13] and postoperative healing problems can occur. The resection of the symphyseal bone mass usually depends on subchondral sclerosis of the symphyseal bone and usually is performed using a chisel until bleeding of the cortical bone [11]. This allows the surgeon to modify the depth of cortical bone resection, but also results in soft tissue damage, especially of the remaining anterior or posterior symphyseal ligaments. To treat osteochondral defects, the usage of press-fit cylinders has been well established in past years [14, 15]. However, its use in the treatment of pelvic non-unions has been described in only few cases. Herath et al. described the usage of Surgical Diamond Instruments (SDI) to create press-fit cylinders for the treatment of posterior pelvic ring non-unions [15]. According to the results of our institution for the posterior pelvic ring, we created the novel minimally-invasive symphysiodesis method as described in this article. Using the cannulated and modified bone graft harvesting tool, a safe resection and harvesting of the symphyseal bone was possible. At the end of the procedure, in the anatomical dissection we found the symphyseal ligaments still completely intact. We estimate that leaving the symphyseal ligaments and performing the symphysiodesis with a press-fit cylinder will result with a high stability of the bone graft and may result in sufficient osseous healing without extensive stabilization as described in previous studies for articular cartilage [8]. In posttraumatic cases or cases with a persisting wideness, reduction of the symphysis, prior to harvesting the symphyseal bone cylinder may help to achieve the same effect with the press-fit bone cylinder transplant. However, in this report we did not analyze any biomechanical or clinical data.

### Limitations

In this study we analyzed the feasibility of a minimally invasive surgical technique for the arthrodesis of the pubic symphysis. So biomechanical stability can only be predicted and has to be analyzed in further studies.

## Conclusion

The described novel minimally invasive surgical technique to create a symphysiodesis in this report, is technically feasible and might be a promising alternative to the more invasive approaches. Furthermore, we also hypothesize, that this novel technique can also be used without additional extensive stabilization in the future, due to the press-fit purchase of the bone graft and its suspected high biomechanical stability.

## Abbreviations

DCP: Dynamic Compression Plate
e.g.: Exempli gratia
SLDCP: Symphyseal Locking Dynamic Compression Plate
3D: Three dimensional
